# Psoriatic arthritis and sacroiliitis are associated with increased vascular inflammation by 18-fluorodeoxyglucose positron emission tomography computed tomography: baseline report from the Psoriasis Atherosclerosis and Cardiometabolic Disease Initiative

**DOI:** 10.1186/ar4676

**Published:** 2014-07-30

**Authors:** Shawn Rose, Jenny Dave, Corina Millo, Haley B Naik, Evan L Siegel, Nehal N Mehta

**Affiliations:** Section of Inflammation and Cardiometabolic Diseases, National Heart, Lung, and Blood Institute (NHLBI), National Institutes of Health, Bethesda, MD 20892 USA; National Institute of Arthritis and Musculoskeletal and Skin Diseases (NIAMS), National Institutes of Health, Bethesda, MD 20892 USA; Nuclear Medicine Positron Emission Tomography Department, National Institutes of Health Clinical Center, Bethesda, MD 20892 USA; National Cancer Institute (NCI), National Institutes of Health, Bethesda, MD 20892 USA; Arthritis and Rheumatism Associate, Rockville, MD 20850 USA

## Abstract

**Introduction:**

Psoriasis and psoriatic arthritis (PsA) increase cardiovascular disease (CVD) risk, but surrogate markers for CVD in these disorders are inadequate. Because the presence of sacroiliitis may portend more severe PsA, we hypothesized that sacroiliitis defined by computed tomography (CT) would be associated with increased vascular inflammation defined by 18-fluorodeoxyglucose-positron emission tomography/computed tomography (FDG-PET/CT), which is an established measure of CVD.

**Methods:**

Participants (*n* = 65) underwent whole-body FDG-PET/CT. Metabolic activity of the aorta was measured using the maximal standardized uptake value (SUV_max_), a measure of atherosclerotic plaque activity. The primary outcome was aortic vascular inflammation. Linear regression (with β-coefficients (β) and *P*-values reported for PsA and sacroiliitis) was used to adjust for CVD risk factors to determine associations of PsA or sacroiliitis with vascular inflammation. Likelihood ratio testing was performed to evaluate the contribution of sacroiliitis to vascular disease estimation compared to the effects of PsA and traditional CVD risk factors.

**Results:**

Vascular inflammation (measured as SUV_max_) was greater (*P* < 0.001) in patients with sacroiliitis (mean ± SD = 7.33 ± 2.09) defined by CT compared to those without sacroiliitis (6.39 ± 1.49, *P* = 0.038). There were associations between PsA and aortic inflammation (β = 0.124, *P* < 0.001) and between sacroiliitis and aortic inflammation (β = 0.270, *P* < 0.001) after adjusting for CVD risk factors. Sacroiliitis predicted vascular inflammation beyond PsA and CVD risk factors (*χ*^2^ = 124.6, *P* < 0.001).

**Conclusions:**

Sacroiliitis is associated with increased vascular inflammation detected by FDG-PET/CT, suggesting that sacroiliac joint disease may identify patients at greater risk for CVD. Large, ongoing prospective studies are required to confirm these findings.

**Electronic supplementary material:**

The online version of this article (doi:10.1186/ar4676) contains supplementary material, which is available to authorized users.

## Introduction

Systemic inflammatory disorders, including psoriasis (Pso) [1–3] and psoriatic arthritis (PsA) [3], increase incident cardiovascular events beyond traditional cardiovascular disease (CVD) risk factors [4]. Understanding of this elevated risk has been hampered by the lack of suitable clinical biomarkers linking systemic inflammation and CVD. Prevention of increased CVD morbidity and mortality in Pso and PsA requires identification of those patients at greatest risk for these diseases prior to the development of overt symptoms. 18-Fluorodeoxyglucose-positron emission tomography/computed tomography (FDG-PET/CT) is an imaging modality that may provide this capability, as it delineates enhanced metabolic activity in tissues *in vivo*, including vascular inflammation [5–9]. Importantly, vascular inflammation seen on FDG-PET/CT scans has been shown to precede the development of atherosclerotic disease [10] and to predict future cardiovascular events [11, 12]. Our prior investigations in which we utilized FDG-PET/CT have shown increased inflammation in multiple tissues, including the skin, liver, joints, entheses and vasculature in Pso and PsA patients compared to healthy controls [6, 13, 14]. We have also previously demonstrated relationships between FDG-PET/CT imaging measurements and both known and novel CVD biomarkers, thereby validating FDG-PET/CT findings as reliable predictors of outcome [15]. However, these pilot studies were underpowered to adequately assess for PsA or axial disease.

Sacroiliitis is a characteristic feature of the spondyloarthropathies, including PsA [16]. Imaging of patients with sacroiliitis is an element of the current classification criteria for these disorders [17, 18]. Imaging of the sacroiliac (SI) joints by CT scan is one modality that is accepted for making the diagnosis of sacroiliitis [19–27]. Further, the presence of sacroiliitis may identify a PsA subgroup at risk for more severe arthropathy [28, 29]. Greater inflammatory burden imposed by severe Pso [1, 30, 31] and the presence of PsA [32, 33] increases the risk of CVD. Thus, we hypothesized that PsA and sacroiliitis diagnosed by CT scan would be associated with increased vascular inflammation by FDG-PET/CT scan in a well-characterized population of Pso patients with versus without a diagnosis of PsA. Here we present a consecutive sample of 65 patients who completed baseline studies in the Psoriasis Atherosclerosis and Cardiometabolic Disease Initiative (PACI; ClinicalTrials.gov Identifier: NCT01778569). A major aim of this prospective, longitudinal study is to identify incident biomarkers of vascular and metabolic disease in Pso and PsA.

## Methods

### Study population

Whole-body FDG-PET/CT was performed in a consecutive sample of patients (*n* = 65) ages 18 to 70 years with Pso alone (*n* = 38) or with both Pso and PsA (*n* = 27). Our study population was recruited to the National Institutes of Health (NIH) Clinical Center using flyers and pamphlets distributed at dermatology clinics and via academic dermatological societies, as well as by using web-driven tools such as [34] and [35]. Diagnostic confirmation of plaque Pso and assessment of body surface area (BSA) and Psoriasis Area and Severity Index (PASI) were performed by a dermatologist. PsA classifications were confirmed by a rheumatologist according to the ClASsification criteria for Psoriatic ARthritis (CASPAR) [36]. A rheumatologist did the swollen and tender joint counts using the American College of Rheumatology 66/68 joint count core set [37]. Clinical assessment of enthesitis (by Leeds Enthesitis Index score [38]), dactylitis (by number of dactylitic digits) and inflammatory back pain (according to Assessment of SpondyloArthritis international Society (ASAS) criteria [39] and Calin criteria [40]) was also performed by a rheumatologist. Exclusion criteria included disease states that can increase systemic or vascular inflammation, such as known history of CVD, uncontrolled hypertension (defined as systolic blood pressure (SBP) >180 mmHg or diastolic blood pressure (DBP) >95 mmHg), nondermatologic malignant disease within the past 5 years, positive HIV status, major surgery within the past 3 months and history of intravenous drug use or active infection within the preceding 72 hours. Hypertension was defined as SBP >140 mmHg, DBP >90 mmHg and/or currently on antihypertensive therapy. Hyperlipidemia was defined as total cholesterol >200 mg/dl, low-density lipoprotein (LDL) ≥160 mg/dl, high-density lipoprotein (HDL) <40 mg/dl, triglycerides >150 mg/dl and/or use of a cholesterol-lowering agent. Diabetes was defined as fasting glucose ≥126 mg/dl, hemoglobin A1c ≥6.5% and/or taking antidiabetic therapy.

### FDG-PET/CT

**Figure 1:**
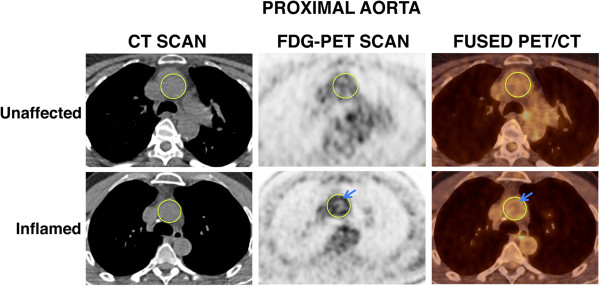
**18-fluorodeoxyglucose-positron emission tomography/computed tomography delineates vascular inflammation.** Images shown are representative computed tomography (CT) (left panels), 18-fluorodeoxyglucose-positron emission tomography (FDG-PET) (center panels) and fused FDG-PET/CT transaxial (right panels) scans from a patient with an unaffected aortic vasculature (upper panel) and one with an inflamed aortic vasculature (lower panel). Regions of interest outlining the ascending thoracic aorta are depicted in yellow in all images. A ring-shaped focus of increased metabolic activity in the aortic wall is also shown (blue arrows in FDG/PET and fused FDG-PET/CT images).

Whole-body FDG-PET/CT scans were obtained using a standardized published protocol [13]. Following an overnight fast, patients were imaged 60 minutes after receiving an injection of 370 MBq of 18-FDG. Images were acquired in three-dimensional mode using a Siemens Biograph TruePoint PET/CT scanner (Siemens Healthcare, Erlangen, Germany). FDG-PET/CT images were reviewed by a reader, blinded to all patient characteristics, using dedicated PET/CT image analysis software (Extended Brilliance Workstation (EBW); Philips Healthcare, Amsterdam, the Netherlands). FDG uptake in the thoracic and abdominal aorta was quantified using published methods [6]. Briefly, circular two-dimensional regions of interest (ROI) circumscribing the external aortic contour were drawn on serial contiguous transaxial 4-mm sections from the level of the aortic root to the iliac bifurcation (Figure 1). Each axial segment provided two measures of tissue metabolic uptake [6]: mean and maximum standardized uptake (SUV_mean_ and SUV_max_, respectively). SUV_mean_ and SUV_max_ values were determined using commercial software (EBW) for each successive slice. SUV_max_ values were averaged over three consecutive slices to measure the most inflamed aortic region in each patient. A nuclear medicine specialist independently evaluated FDG-PET/CT scans for imaging quality, radiotracer biodistribution, uptake time and clinical findings.

### Imaging evaluation for sacroiliitis

Sacroiliitis diagnosed by CT scan was evaluated using guidelines developed by Geijer and colleagues [41]. Sacroiliitis grade was classified as “no disease” (that is, radiographically normal, degenerative change evident or some blurring of the joint margins), “suspected disease” (that is, unilateral sclerosis with some erosion) or “definite disease” (that is, bilateral sclerosis with erosion, or unilateral or bilateral sclerosis with severe erosion and a change in joint space with or without partial ankylosis, or complete ankylosis). Both axial and coronal CT scan orientations were examined. Sclerosis, erosions, ankylosis and/or joint space changes had to be evident on at least two consecutive slices to be deemed significant.

### Serum factor determination

Fasting levels of total, HDL and LDL cholesterol, triglycerides, glucose, erythrocyte sedimentation rate (ESR) and high-sensitivity C-reactive protein (hsCRP) were measured in a clinical laboratory.

### Statistical analysis

Normality was assessed by skewness and kurtosis. Normally distributed continuous variables were compared using Student’s *t*-test. Continuous variables lacking a normal distribution were compared using Mann–Whitney *U* test. Dichotomous variable comparisons were performed using Fisher’s exact test. Student’s *t*-test was employed to compare FDG-PET/CT measures (SUV_max_) of aortic inflammation between Pso and PsA groups and between patients with or without CT evidence of sacroiliitis. Only those patients with definitive disease defined by CT were considered to have sacroiliitis for all analyses. Patients not meeting the CASPAR criteria [36] for PsA were classified as having PSO. Relationships between variables were determined using Spearman correlation analysis and are reported as Spearman ρ (*r*) values. Multivariate linear regression analysis was performed using aortic SUV_max_ as the dependent variable and CVD risk factors (age, sex, body mass index (BMI), diabetes, hypertension, dyslipidemia and pack-years of tobacco use) and either PsA or sacroiliitis evident by CT scan as independent variables, as reported. Sex, diabetes, hypertension, dyslipidemia, PsA and sacroiliitis were adjusted for as dichotomous variables in the models; all other variables were continuous. Similar findings were revealed in independent analyses designating sex, PsA and sacroiliitis as dichotomous variables and age, BMI, fasting glucose, SBP, LDL cholesterol, tobacco use and aortic SUV_max_ as continuous variables. Both fixed- and random-effects regression analyses were performed on each model to accommodate within-patient correlation of SUV_max_ values [13]. Because the magnitude of the estimates of PsA and sacroiliitis using vascular measures did not differ when fixed- and random-effects regression analyses were performed, we report β-coefficients (β) and *P*-values for the fixed effects models after adjustment for CVD risk factors. Further, we examined the outcome variable aortic SUV_max_ in separate multivariate linear regression models using either slice-by-slice SUV_max_ measures or collapsed individual patient values. The magnitude of the effect of the covariates on aortic SUV_max_ was similar in both models; hence, we report the slice-by-slice data. On the basis of prior published results [5] demonstrating that aortic SUV increased 0.1 U (SD ±0.1) per decade of life, we considered this to be a clinically relevant difference. Therefore, with these assumptions, our sample size (*n* = 65) provided >90% power to detect a difference of 0.1 in aortic vascular inflammation by the presence or absence of SI inflammation. Likelihood-ratio testing was performed in nested Tobit models to determine the incremental value of sacroiliitis to CVD estimation above and beyond PsA and traditional CVD risk factors. STATA 12 statistical software (StataCorp, College Station, TX, USA) was utilized for all analyses. Study approval was obtained from the National Heart, Lung, and Blood Institute Institutional Review Board in accordance with the Declaration of Helsinki. All guidelines for GCP and those set forth by the NIH Radiation Safety Commission and in the Belmont Report (National Commission for the Protection of Human Subjects of Biomedical and Behavioral Research) were followed. All study participants provided written informed consent.

## Results

### Patient demographics, disease assessments and CVD risk factors

Patient characteristics (*n* = 65) and laboratory measurements are presented in Table 1 for the whole sample and for the Pso (*n* = 38) and PsA (*n* = 27) subgroups. Our study population had a median age of 53 years, was nearly equally distributed by sex (54% male) and had moderate skin disease on average (mean BSA = 9.2, mean PASI score = 7.8) (Table 1). The proportion who had PsA was 41%. Median skin disease duration was 20 years for both the Pso and PsA subgroups, and the median duration of joint disease was 10 years in PsA patients (Table 1). One patient met both the Calin and ASAS criteria for inflammatory back pain. This patient was classified as having Pso, however, due to not meeting the CASPAR criteria for PsA and having CT images showing advanced degenerative and postsurgical changes in the spine without evidence of spondylitis or sacroiliitis. Two additional patients were definitively diagnosed with sacroiliitis by CT without evidence of inflammatory back pain, SI tenderness to palpation or provocative maneuvers, arthritis, enthesitis or dactylitis. Because both of those two patients met the CASPAR criteria for PsA, they were classified as having asymptomatic axial PsA. Sacroiliitis diagnosed by CT scan was confirmed in 44% of PsA patients (Table 1).

**Table 1:** **Patient characteristics** ^**a**^

Characteristics	All ( *n* = 65)	Pso ( *n* = 38)	PsA ( *n* = 27)	*P* -value
**Age, yr**	53 (41 to 61)	54 (41 to 65)	52 (41 to 59)	0.526
**Males,** ***n*** **(%)**	35 (54)	21 (55)	14 (52)	0.806
**Psoriasis disease duration, yr**	20 (9 to 32)	20 (8 to 35)	20 (10 to 31)	0.931
**Body surface area score, mean (SD)**	9.2 (16)	8.1 (13.7)	10.8 (19.0)	0.827
**PASI score, mean (SD)**	7.8 (9.3)	7.3 (7.9)	8.7 (11.2)	0.967
**Psoriatic arthritis,** ***n*** **(%)**	27 (41)	–	–	–
**Psoriatic arthritis disease duration, yr**	–	–	10 (4 to 15.5)	–
**Tender joint count (0 to 68), mean (SD)**	–	–	8.9 (10.8)	–
**Swollen joint count (0 to 66), mean (SD)**	–	–	5.4 (9.0)	**–**
**ASAS inflammatory back pain criteria met,** ***n*** **(%)**	–	–	4 (17)	–
**Calin inflammatory back pain criteria met,** ***n*** **(%)**	–	–	5 (21)	–
**Enthesitis score (0 to 6), mean (SD)**	–	–	1.0 (1.5)	**–**
**Dactylitis count (0 to 20), mean (SD)**	–	–	1.4 (3.8)	**–**
**Sacroiliac disease diagnosed by CT,** ***n*** **(%)**	–	–	12 (44)	–
**DMARD therapy,** ***n*** **(%)**	6 (9)	2 (5)	4 (15)	0.224
**Biologic therapy,** ***n*** **(%)**	25 (39)	10 (26)	15 (56)	**0.022**
**DMARD and biologic therapy,** ***n*** **(%)**	4 (6)	2 (5)	2 (7)	1.000
**NSAID therapy,** ***n*** **(%)**	15 (23)	3 (8)	12 (44)	**0.001**
**Phototherapy,** ***n*** **(%)**	3 (5)	1 (3)	2 (7)	0.565
**Topical steroid therapy,** ***n*** **(%)**	24 (37)	14 (37)	10 (37)	1.000
**Systemic steroid therapy,** ***n*** **(%)**	1 (2)	0 (0)	1 (4)	0.415
**Diabetes mellitus,** ***n*** **(%)**	7 (11)	4 (11)	3 (11)	1.000
**Hypertension,** ***n*** **(%)**	21 (32)	13 (34)	8 (30)	0.791
**Dyslipidemia,** ***n*** **(%)**	44 (68)	29 (76)	15 (56)	0.108
**Current tobacco use,** ***n*** **(%)**	6 (9)	3 (8)	3 (11)	1.000
**Former tobacco use,** ***n*** **(%)**	18 (28)	12 (32)	6 (22)	0.684
**Pack-years tobacco use**	0 (0 to 4.5)	0 (0 to 5)	0 (0 to 0.8)	0.590
**Diabetes mellitus therapy,** ***n*** **(%)**	4 (6)	2 (5)	2 (7)	1.000
**Antihypertensive therapy,** ***n*** **(%)**	12 (19)	7 (18)	5 (19)	1.000
**Hyperlipidemia therapy,** ***n*** **(%)**	24 (37)	15 (39)	9 (33)	0.795
**Body mass index, kg/m** ^**2**^	29 (25.9 to 32.3)	29 (24.9 to 32.4)	29 (26.7 to 32)	0.572
**Systolic blood pressure, mmHg**	125 (116 to 135)	129 (120 to 135)	122 (115 to 133)	0.266
**Diastolic blood pressure, mmHg**	72 (65 to 78)	71 (66 to 78)	74 (63 to 79)	0.719
**Fasting blood glucose, mg/dl**	94 (89 to 104)	94 (89 to 104)	94 (89 to 107)	0.895
**Total cholesterol, mg/dl**	184 (158 to 203)	185 (158 to 207)	172 (157 to 201)	0.910
**Triglycerides, mg/dl**	108 (84 to 137)	109 (84 to 149)	101 (81 to 131)	0.604
**High-density lipoprotein cholesterol, mg/dl**	52 (42 to 63)	52 (42 to 69)	51 (47 to 59)	0.947
**Low-density lipoprotein cholesterol, mg/dl**	96 (80 to 125)	95 (80 to 125)	98 (78 to 125)	0.851
**Erythrocyte sedimentation rate, mm/hr**	8 (5 to 13)	8 (4 to 10)	12 (5 to 18)	0.097
**High-sensitivity C-reactive protein, g/dl**	1.7 (0.7 to 4.2)	1.2 (0.7 to 3.1)	3.0 (0.8 to 8)	0.221

^a^ASAS, Assessment of SpondyloArthritis international Society; DMARD, Disease-modifying antirheumatic drug; IQR, Interquartile range; NSAID, Nonsteroidal anti-inflammatory drug; PASI, Psoriasis Area and Severity Index; PsA, Psoriatic arthritis; Pso, Psoriasis. Data are reported as median (IQR) unless indicated otherwise. DMARD therapy denotes methotrexate use, except for 1 patient who was taking both methotrexate and hydroxychloroquine. Biologic therapy indicates active TNF antagonist or anti-IL-12/23 receptor use except for one patient who was treated with abatacept for psoriatic arthritis. Reported *P*-values are for comparisons of Pso vs. PsA using Fisher’s exact tests for categorical variables, Mann-Whitney U tests for non-normally distributed continuous variables, and Student’s t-tests for normally distributed continuous variables. *P* < 0.05 was set as the significance level.

We also assessed Medication use and cardiovascular and metabolic comorbidities in our study population (Table 1). Many patients (37%) were using topical steroids, whereas systemic steroid therapy and phototherapy were rare (Table 1). Disease-modifying antirheumatic drug (DMARD) treatment was uncommon and was not statistically different (*P* = 0.224) between the PsA (15%) and Pso (5%) groups (Table 1). Biologic therapy was more common (*P* = 0.022) in PsA patients (56%) than in Pso patients (26%). Traditional CVD risk factors, including hypertension, dyslipidemia, diabetes mellitus and tobacco use, were prevalent in our study population, as was treatment for these disorders. Presence and treatment of CVD risk factors were not statistically different between the Pso and PsA subgroups (Table 1).

### Vascular inflammation measured by FDG-PET/CT is greater in patients with sacroiliitis defined by CT

We utilized FDG-PET/CT to examine vascular inflammation, as defined by aortic SUV_max_, in our study population. Mean (SD) SUV_max_ values for the aortic region demonstrating the highest vascular inflammation in three contiguous slices were numerically, but not statistically, greater (*P* = 0.536) in PsA patients (6.72 ± 1.92) compared to Pso patients (6.46 ± 1.43) (Table 2). Because sacroiliitis has been shown to signify worse arthropathy in PsA [28, 29], we hypothesized that the greater inflammatory state in patients with sacroiliitis may lead to increased vascular inflammation visualized by FDG-PET/CT. Indeed, mean aortic SUV_max_ measures (Table 2) were significantly greater (*P* = 0.038) in patients with CT evidence of sacroiliitis (7.33 ± 2.09) compared to those without SI joint disease (6.39 ± 1.49). Further, aortic vascular inflammation was greatest in those demonstrating the highest grade (definitive disease) of sacroiliitis by CT scan (Additional file 1: Table S1).

**Table 2:** **Vascular inflammation is increased in patients with sacroiliitis** ^**a**^

	Pso ( *n* = 38)	PsA ( *n* = 27)	
	**Mean ± SD**	**Mean ± SD**	***P*** **-value**
**Aortic SUV**_**max**_	6.46 ± 1.43	6.72 ± 1.92	0.536
	Sacroiliitis absent (*n* = 53)	Sacroiliitis present (*n* = 12)	
	**Mean ± SD**	**Mean ± SD**	***P*** **-value**
**Aortic SUV**_**max**_	6.39 ± 1.49	7.33 ± 2.09	**0.038**

^a^CT, Computed tomography; PsA, Psoriatic arthritis; Pso, Psoriasis; SUV_max_, Maximum standardized uptake value. Data are reported as mean ± SD of the greatest aortic SUV_max_ region (three consecutive slices) for each patient. Indicated *P*-values (*P* < 0.05 deemed significant) are for Student’s *t*-test comparisons of either Pso vs PsA or absence vs presence of sacroiliitis by CT scan.

### Sacroiliitis defined by CT scan correlates with vascular inflammation evaluated by FDG-PET/CT above and beyond CVD risk factors

To determine covariates of vascular inflammation by highest atherosclerotic plaque activity within our cohort, individual Spearman correlation analyses were performed between aortic SUV_max_ and clinical, laboratory and imaging parameters in the total population and in the Pso and PsA subgroups (Additional file 2: Table S2). In unadjusted analyses of the total study population (Additional file 2: Table S2), aortic SUV_max_ was significantly related to traditional CVD risk factors, including age (*r* = 0.406, *P* < 0.001), sex (*r* = 0.146, *P* < 0.001), BMI (*r* = 0.507, *P* < 0.001), hypertension (*r* = 0.204, *P* < 0.001), diabetes (*r* = 0.186, *P* < 0.001), dyslipidemia (*r* = 0.226, *P* < 0.001) and tobacco use (*r* = -0.021, *P* = 0.01). Sacroiliitis (*r* = 0.213, *P* < 0.001) was more strongly related to vascular inflammation than were the relationships between vascular inflammation and psoriatic arthritis (*r* = 0.033, *P* < 0.001), PASI score (*r* = 0.001, *P* = 0.919), ESR (*r* = 0.061, *P* < 0.001) or hsCRP (*r* = -0.005, *P* = 0.513) in the total sample. In general, aortic SUV_max_ and CVD risk factors were more strongly related in PsA than in Pso patients (Additional file 2: Table S2). When these analyses were performed using SUV_mean_ as the outcome variable, similar results were obtained. The aortic SUV_max_ data are reported, as this measurement indicates the region of most intense vascular inflammation.

In multivariate regression analyses (Table 3), sacroiliitis remained highly associated with vascular inflammation, even after adjusting for age, sex and BMI (β = 0.268, *P* < 0.001) and with additional adjustment for hypertension, dyslipidemia, diabetes and tobacco use (β = 0.270, *P* < 0.001). PsA was also associated with vascular inflammation (Table 3) in both partially adjusted (age, sex and BMI; β = 0.117, *P* < 0.001) and fully adjusted models (age, sex, BMI, hypertension, dyslipidemia, diabetes and tobacco; β = 0.124, *P* < 0.001). To obtain a crude estimate for systemic inflammatory burden, multivariate regression analyses were further adjusted for ESR and hsCRP levels. Addition of ESR (β = 0.268, *P* < 0.001) or hsCRP (β = 0.303, *P* < 0.001) as independent variables did not affect the relationship between sacroiliitis and vascular inflammation in fully adjusted models (Additional file 3: Table S3). PsA and aortic SUV_max_ also remained significantly related after adding ESR (β = 0.110, *P* < 0.001) or hsCRP (β = 0.133, *P* < 0.001) into fully adjusted models. The contribution of sacroiliitis to aortic SUV_max_ estimation beyond PsA and/or traditional CVD risk factors was determined using likelihood-ratio testing (Table 4). Sacroiliitis defined by CT scan predicted vascular inflammation (Table 4) above and beyond PsA in unadjusted models (*χ*^2^ = 374.6, *P* < 0.001) and beyond PsA and CVD risk factors in partially adjusted (*χ*^2^ = 132.8, *P* < 0.001) and fully adjusted models (*χ*^2^ = 124.6, *P* < 0.001). Together, these findings suggest that sacroiliitis defined by CT scan may independently confer risk of higher vascular inflammation in patients with Pso and PsA.

**Table 3:** **Sacroiliitis and psoriatic arthritis are positively related to vascular inflammation even after adjustment for CVD risk factors** ^**a**^

Regression factors	Age, sex and BMI	Age, sex, BMI, HTN, DL, DM, Tob
Sacroiliitis	0.268 (*P* < 0.001)	0.270 (*P* < 0.001)
Psoriatic arthritis	0.117 (*P* < 0.001)	0.124 (*P* < 0.001)

^a^Multivariate linear regression analyses were performed using aortic maximum standardized uptake (SUV_max_) as the dependent variable. Independent variables included in the model were (1) age, sex, body mass index (BMI) and either sacroiliitis (row 1, left column) or psoriatic arthritis (row 2, left column) or (2) age, sex, BMI, hypertension (HTN), dyslipidemia (DL), diabetes mellitus (DM), tobacco use (Tob) and either sacroiliitis (row 1, right column) or psoriatic arthritis (row 2, right column). β-coefficients (*P*-values) are reported for the effects of sacroiliitis or psoriatic arthritis on vascular inflammation (aortic SUV_max_) after adjustment for cardiovascular disease risk factors (age, sex, BMI, HTN, DL, DM and Tob). *P*-values <0.05 were considered significant.

**Table 4:** **Sacroiliitis predicts vascular inflammation beyond psoriatic arthritis and CVD risk factors** ^**a**^

Model	Aortic SUV_max_ *χ* ^2^(*P*-value)
**Unadjusted**	374.6 (<0.001)
**Partially adjusted**	132.8 (<0.001)
**Fully adjusted**	124.6 (<0.001)

^a^Likelihood ratio testing was applied in nested Tobit models to assess the incremental value of sacroiliitis in predicting aortic vascular inflammation. The outcome variable was aortic maximum standardized uptake (SUV_max_). Independent variables in the different models were as follows: psoriatic arthritis (unadjusted); age, sex, BMI and psoriatic arthritis (partially adjusted); and age, sex, BMI, hypertension, dyslipidemia, diabetes, tobacco use and psoriatic arthritis (fully adjusted). *χ* ^2^ values are reported for each model. *P*-values < 0.05 were considered significant.

## Discussion

To our knowledge, this report is the first to link sacroiliitis defined by CT scan and vascular inflammation in Pso and PsA, an association that persisted after adjustment for traditional CVD risk factors. Aortic inflammation measured by FDG-PET/CT was found to be greater in patients with evidence of sacroiliitis compared to those without SI joint disease. We were able to determine that sacroiliitis impacted vascular inflammation in a dose-dependent fashion, as aortic SUV_max_ values were greatest in those with sacroiliitis definitively diagnosed by CT scan compared to those with no disease or suspected disease. Further, vascular inflammation measured by FDG-PET/CT correlated with sacroiliitis in both unadjusted models and in models adjusted for CVD risk factors and markers of systemic inflammation. Importantly, sacroiliitis was a strong predictor of CVD above and beyond PsA and traditional risk factors. Taken together, these findings suggest that sacroiliitis not only may identify those at risk for more severe joint disease [28, 29] but may also be an indicator of increased susceptibility to CVD.

Identification of sacroiliitis as a correlate of CVD holds tremendous clinical promise since it is a characteristic feature of PsA [17, 18] and can readily be screened for in this disorder. Since use of FDG-PET/CT for CVD risk stratification in Pso and PsA is unlikely to extend beyond the research realm in the foreseeable future, sacroiliitis could potentially serve as a marker of increased risk of CVD in these populations. Future studies should extend our findings by examining the relationship between sacroiliitis and CVD in other spondyloarthropathies, and by determining whether active inflammation in the SI joint confers even greater risk for CVD. Whether sacroiliitis also relates to other psoriatic comorbidities is a subject of ongoing investigation in our cohort.

It has been suggested that higher inflammatory burden imparts increased CVD risk in the systemic inflammatory diseases [4]. Indeed, greater skin disease severity has been linked to increased CVD mortality in Pso [1, 30, 31, 42], and this observation has been further supported by data demonstrating cardiometabolic and CVD modulation in lesional psoriatic skin [43, 44]. Furthermore, PsA has generally been considered to represent a heightened inflammatory state compared to Pso alone [3], and thus PsA may confer an elevated risk of CVD-associated morbidity and mortality [45]. Although epidemiologic studies have yielded conflicting data regarding all-cause mortality risk in PsA [42, 46–51], the results of several studies demonstrate that PsA increases CVD-attributable mortality compared to the general population [30, 42, 52] and to Pso patients [32, 33, 42, 53]. Our findings suggest that screening for particular features of Pso and PsA rather than global measures of systemic inflammation may be a more viable strategy for identifying patients at increased risk for CVD. In support of this notion, our results presented here demonstrate that sacroiliitis, as defined by CT scan, and vascular inflammation were associated with PsA above and beyond traditional CVD risk factors and independent of levels of systemic inflammatory markers (ESR and hsCRP). Thus, sacroiliitis may be a potentially useful metric of CVD beyond crude measures of systemic inflammation.

We acknowledge that this study has certain limitations and that additional research is required to corroborate and extend our findings. The modest sample size and single-center study design could affect the generalizability of the results. Further, FDG-PET/CT may not be suitable for widespread adoption, owing to its limited availability for nonmalignant conditions and its mild radiation exposure. In addition, evaluation of sacroiliitis by CT scan may overestimate the frequency of patients with sacroiliitis [41, 54], which could have impacted our results. Although analyses were adjusted for CVD risk factors and markers of systemic inflammation, other potential confounding variables, including Pso and PsA disease duration as well as disease activity and therapy, may have decreased the magnitude of the observed associations. Our study design was generally inclusive in order to allow us to understand the impact of Pso and PsA on vascular inflammation across a wide range of disease manifestations and therapies (that is, in a setting closer to the “real world”). Thus, the relationship between sacroiliitis and vascular inflammation may have been underestimated, as no washout medication period was required prior to study enrollment. However, it is remarkable that, though many of the Pso and PsA patients were undergoing treatment to mitigate their inflammatory disease and/or CVD risk factors, we found that sacroiliitis and vascular inflammation were still readily detectible.

Despite these limitations, our data implicate sacroiliitis as a significant and novel marker of increased CVD risk in PsA. Further, FDG-PET/CT may hold promise to improve CVD risk estimation as well as to delineate tissue inflammation in the rheumatic diseases. The PACI study (ClinicalTrials.gov Identifier: NCT01778569) is uniquely poised to address these questions, as it includes a large, ongoing, longitudinal cohort in which serial FDG-PET/CT scans will be utilized to detect incident sacroiliitis and CVD in Pso and PsA patients. Additional studies are needed to substantiate and expand our present findings, to link them to clinical prognoses and to understand the effects of treatment on tissue-specific inflammation in Pso and PsA patients.

## Conclusion

Sacroiliitis is associated with increased vascular inflammation beyond traditional CVD risk factors, suggesting that sacroiliitis may identify patients at greater risk for vascular complications.

## Authors’ information

SR is an American Board of Internal Medicine–certified rheumatologist, a member of the Group for the Assessment of Psoriasis and Psoriatic Arthritis (GRAPPA) and a Metzger Clinical Scholar at the National Institutes of Health. SR has extensive expertise in the clinical, basic and translational mechanisms linking inflammatory disorders to cardiovascular and metabolic disease. JD is an Intramural Research Training Awardee (IRTA) at the National Institutes of Health who specializes in multimodal imaging analyses. CM is an American Board of Radiology–certified radiologist and nuclear medicine specialist who works at the National Institutes of Health Clinical Center. HN is an American Board of Dermatology–certified dermatologist with extensive expertise in psoriasis, graft-versus-host disease and the neutrophilic dermatoses, as well as a senior clinical scholar at the National Cancer Institute. ELS is an American Board of Internal Medicine–certified rheumatologist who practices at the Arthritis and Rheumatism Associates, which is the largest rheumatology practice in the Washington, DC, Virginia and Maryland area. He is also a member of the Group for the Assessment of Psoriasis and Psoriatic Arthritis (GRAPPA). NNM is an American Board of Internal Medicine–certified cardiologist who specializes in preventative cardiology and cardiovascular imaging. He is the inaugural Lasker Clinical Scholar and section chief in the Section of Inflammation and Cardiometabolic Diseases at the National Heart, Lung, and Blood Institute, National Institutes of Health. He is also a member of Group for the Assessment of Psoriasis and Psoriatic Arthritis (GRAPPA) and the founder of the Psoriasis, Atherosclerosis, and Cardiometabolic Disease Initiative (ClinicalTrials.gov Identifier: NCT01778569).

## Electronic supplementary material

Additional file 1: Table S1: Aortic vascular inflammation is greater in patients with the highest grade sacroiliitis. These data demonstrate that those patients with definitive sacroiliitis on CT scan demonstrate greater vascular inflammation by FDG-PET/CT compared to patients with suspected or no sacroiliac disease. (TIFF 611 KB)

Additional file 2: Table S2: Correlation analysis of aortic vascular inflammation with clinical measures by subgroup. These data demonstrate that cardiovascular risk factors, sacroiliitis by CT scan and psoriatic arthritis are independently associated with vascular inflammation by FDG-PET/CT. (TIFF 608 KB)

Additional file 3: Table S3: Sacroiliitis and psoriatic arthritis are positively related to vascular inflammation even after adjustment for cardiovascular disease risk factors and the markers of systemic inflammation ESR and CRP. These data demonstrate that sacroiliitis by CT scan and psoriatic arthritis are associated with vascular inflammation by FDG-PET/CT beyond traditional cardiovascular risk factors and circulating markers of systemic inflammation. (TIFF 932 KB)

## Abbreviations

CVD: Cardiovascular disease
DBP: Diastolic blood pressure
DMARD: Disease-modifying antirheumatic drug
ESR: Erythrocyte sedimentation rate
FDG-PET/CT: 18-Fluorodeoxyglucose-positron emission tomography/computed tomography
HDL: High-density lipoprotein
hsCRP: High-sensitivity C-reactive protein
LDL: Low-density lipoprotein
PASI: Psoriasis Area and Severity Index
PsA: Psoriatic arthritis
Pso: Psoriasis
ROI: Region of interest
SBP: Systolic blood pressure
SI: Sacroiliac
SUV: Standardized uptake value.
