# Supplementary figures and images for: Psoriatic arthritis and sacroiliitis are associated with increased vascular inflammation by 18-fluorodeoxyglucose positron emission tomography computed tomography: baseline report from the Psoriasis Atherosclerosis and Cardiometabolic Disease Initiative
Source: Arthritis Res Ther. 2014 Jul 30;16(4):R161. doi: 10.1186/ar4676 (PMC4261785; doi:10.1186/ar4676)

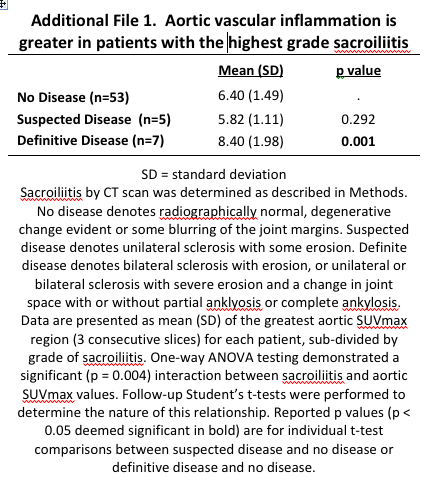

Supplement: Supplementary file 1 — Additional file 1: Table S1: Aortic vascular inflammation is greater in patients with the highest grade sacroiliitis. These data demonstrate that those patients with definitive sacroiliitis on CT scan demonstrate greater vascular inflammation by FDG-PET/CT compared to patients with suspected or no sacroiliac disease. (TIFF 611 KB) [file 13075_2014_4350_MOESM1_ESM.tiff]

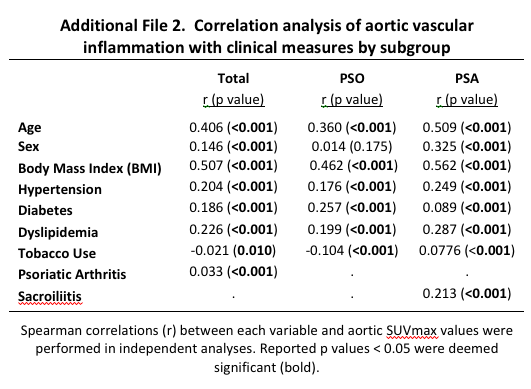

Supplement: Supplementary file 2 — Additional file 2: Table S2: Correlation analysis of aortic vascular inflammation with clinical measures by subgroup. These data demonstrate that cardiovascular risk factors, sacroiliitis by CT scan and psoriatic arthritis are independently associated with vascular inflammation by FDG-PET/CT. (TIFF 608 KB) [file 13075_2014_4350_MOESM2_ESM.tiff]

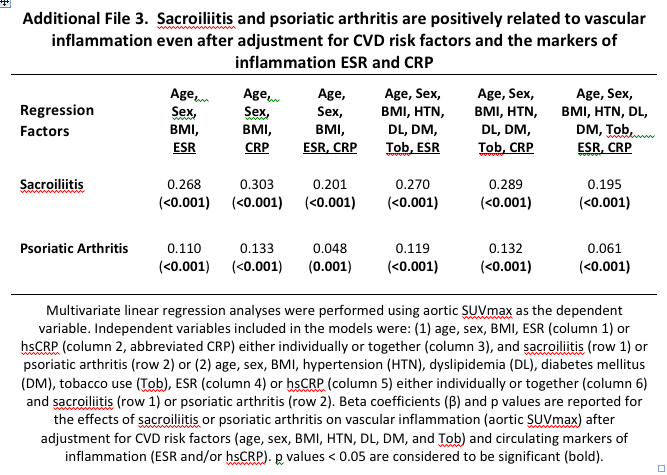

Supplement: Supplementary file 3 — Additional file 3: Table S3: Sacroiliitis and psoriatic arthritis are positively related to vascular inflammation even after adjustment for cardiovascular disease risk factors and the markers of systemic inflammation ESR and CRP. These data demonstrate that sacroiliitis by CT scan and psoriatic arthritis are associated with vascular inflammation by FDG-PET/CT beyond traditional cardiovascular risk factors and circulating markers of systemic inflammation. (TIFF 932 KB) [file 13075_2014_4350_MOESM3_ESM.tiff]
